# Distribution and source identification of dissolved sulfate by dual isotopes in waters of the Babu subterranean river basin, SW China

**DOI:** 10.1007/s10967-017-5217-y

**Published:** 2017-03-16

**Authors:** Kun Ren, Xiaodong Pan, Jie Zeng, Youjun Jiao

**Affiliations:** 1 Institute of Karst Geology, Chinese Academy of Geological Sciences, No. 50, Qixing Road, Guilin, 541004 Guangxi People’s Republic of China; 2 Karst Dynamics Laboratory, Ministry of Land and Resources, Guilin, 541004 Guangxi China

**Keywords:** Dissolved sulfate, Sulfur and oxygen isotopes, Karst, Source identification, Babu subterranean river basin

## Abstract

Sulfur and oxygen isotopes were employed to identify SO_4_^2−^ sources in surface water and groundwater in the Babu subterranean river basin (BSRB). Our study revealed SO_4_^2−^ enrichment in the BSRB waters compared with adjacent areas. The SO_4_^2−^ in some samples originated mainly from precipitation; in others, it was derived mainly from sulfide dissolution in coal seams or from gypsum dissolution. In the water at the subterranean river exit, 13% of SO_4_^2−^ originated from precipitation, 40% from sulfide oxidation in coal seams, and 47% from gypsum dissolution.

## Introduction

Dissolved sulfate (SO_4_^2−^) is not only an important component in water but it also affects acidification, mineral content, and water quality [1–3]. In groundwater, SO_4_^2−^ originates mainly from the dissolution of sulfate-bearing rocks, oxidation of sulfide minerals, and human activities [4, 5]. Because SO_4_^2−^ from different sources is characterized by different “fingerprints,” $\delta^{34} {\text{S}}_{{{\text{SO}}_{4} }}$ has been used widely to track the sources of SO_4_^2−^ in water [6–10]. However, using $\delta^{ 3 4} {\text{S}}_{{{\text{SO}}_{4} }}$ alone to track the source of SO_4_^2−^ in water has two major limitations. The first is that the $\delta^{ 3 4} {\text{S}}_{{{\text{SO}}_{4} }}$ values in precipitation (<+10‰) are within a range that overlaps those produced by oxidized sulfides (<+5‰), causing tremendous difficulties in distinguishing the two. The second limitation is that $\delta^{ 3 4} {\text{S}}_{{{\text{SO}}_{4} }}$ values increase because of reduction by sulfate-reducing bacteria, a characteristic that is indistinguishable from the $\delta^{ 3 4} {\text{S}}_{{{\text{SO}}_{4} }}$ signal caused by gypsum dissolution (>+15‰) [11, 12]. However, the oxygen ($\delta^{ 1 8} {\text{O}}_{{{\text{SO}}_{4} }}$) isotope values of precipitation are relatively high (approximately +12‰) [13], ranging from −5 to +4‰ in oxidized sulfides [14] and from +14.5 to +32.5‰ in gypsum [15, 16]. Therefore, combined use of $\delta^{ 3 4} {\text{S}}_{{{\text{SO}}_{4} }}$ and $\delta^{ 1 8} {\text{O}}_{{{\text{SO}}_{4} }}$ can overcome the problem of $\delta^{ 3 4} {\text{S}}_{{{\text{SO}}_{4} }}$ overlap from different sources and help identify the source of SO_4_^2−^ in water bodies. Hosono et al. [11] analyzed the $\delta^{ 3 4} {\text{S}}_{{{\text{SO}}_{4} }}$ and $\delta^{ 1 8} {\text{O}}_{{{\text{SO}}_{4} }}$ compositions of groundwater in Manila, the capital of the Philippines, and they found artificial chemical compounds (such as sulfur-containing chemical fertilizers and detergents) in shallow groundwater. Li et al. [1] used $\delta^{ 3 4} {\text{S}}_{{{\text{SO}}_{4} }}$ and $\delta^{ 1 8} {\text{O}}_{{{\text{SO}}_{4} }}$ to identify the source of SO_4_^2−^ in the Jialing River, a tributary of the Yangtze River in China. They revealed that the main source of SO_4_^2−^ in the river is acid rain caused by oxidation of sulfides and coal burning during the wet season, while domestic sewage and industrial wastewater contribute more significantly to the SO_4_^2−^ content during the dry season. Using both $\delta^{ 3 4} {\text{S}}_{{{\text{SO}}_{4} }}$ and $\delta^{ 1 8} {\text{O}}_{{{\text{SO}}_{4} }}$, Zhang et al. [12] found that SO_4_^2−^ in the Yellow River (China) and its tributaries originates from dissolved evaporite minerals and soil sulfates, with additional SO_4_^2−^ input by human activities. Marques et al. [2] combined $\delta^{ 3 4} {\text{S}}_{{{\text{SO}}_{4} }}$ and $\delta^{ 1 8} {\text{O}}_{{{\text{SO}}_{4} }}$ to identify the source of SO_4_^2−^ in groundwater. They found that SO_4_^2−^ in groundwater in the Caldas da Rainha area in Portugal originated mainly from dissolved gypsum and anhydrite. Using the same approach, Al-Charideh et al. [17] identified gypsum dissolution as the main source of SO_4_^2−^ in a deep karst aquifer in the Aleppo Basin in northern Syria.

Groundwater in karst areas is an important water resource. Approximately 20–25% of the world’s population use groundwater from karst areas as drinking water [18]. However, pollutants can penetrate into underground aquifers directly or indirectly through thin soil layers, sinkholes, karst windows, and karst fissures. In addition, the poor self-purification ability of aquifers in karst areas makes groundwater in such areas vulnerable to pollution and difficult to restore once polluted [19–21]. Therefore, it is very important to identify accurately the source of pollutants in surface water and groundwater in karst areas. A hydrogeological and geo-environmental survey conducted in Guizhou Province of southwestern China in 2012 revealed that the SO_4_^2−^ concentration in the Babu subterranean river basin (BSRB) (surface water and groundwater) was >50 mg L^−1^ with a peak of up to 1959.8 mg L^−1^, significantly exceeding the drinking water standards in China (250 mg L^−1^). Nevertheless, groundwater remains the principal source of drinking water for residents in this area; in particular, it is the only source of drinking water during the dry season. Long-term consumption of water with such a high SO_4_^2−^ content inevitably endangers human health, causing illnesses such as diarrhea, dehydration, and gastrointestinal disorders.

This study focused on the BSRB in SW China. It examined the surface water and groundwater as carriers and analyzed $\delta^{ 3 4} {\text{S}}_{{{\text{SO}}_{4} }}$ and $\delta^{ 1 8} {\text{O}}_{{{\text{SO}}_{4} }}$ to accomplish a number of objectives: (1) to find the distribution characteristics of SO_4_^2−^ in rainwater, surface water, and groundwater; (2) to identify the sources of SO_4_^2−^ in surface water and groundwater; and (3) to elucidate the contributions of different sources to the SO_4_^2−^ content of the Babu subterranean river. The aims of this study were to provide reference scientific data to enable the development of an effective strategy for the reduction of inputs of SO_4_^2−^ from different sources, and to find an appropriate balance between economic development and the preservation of water quality in karst areas.

## Overview of the study area

**Fig. 1:**
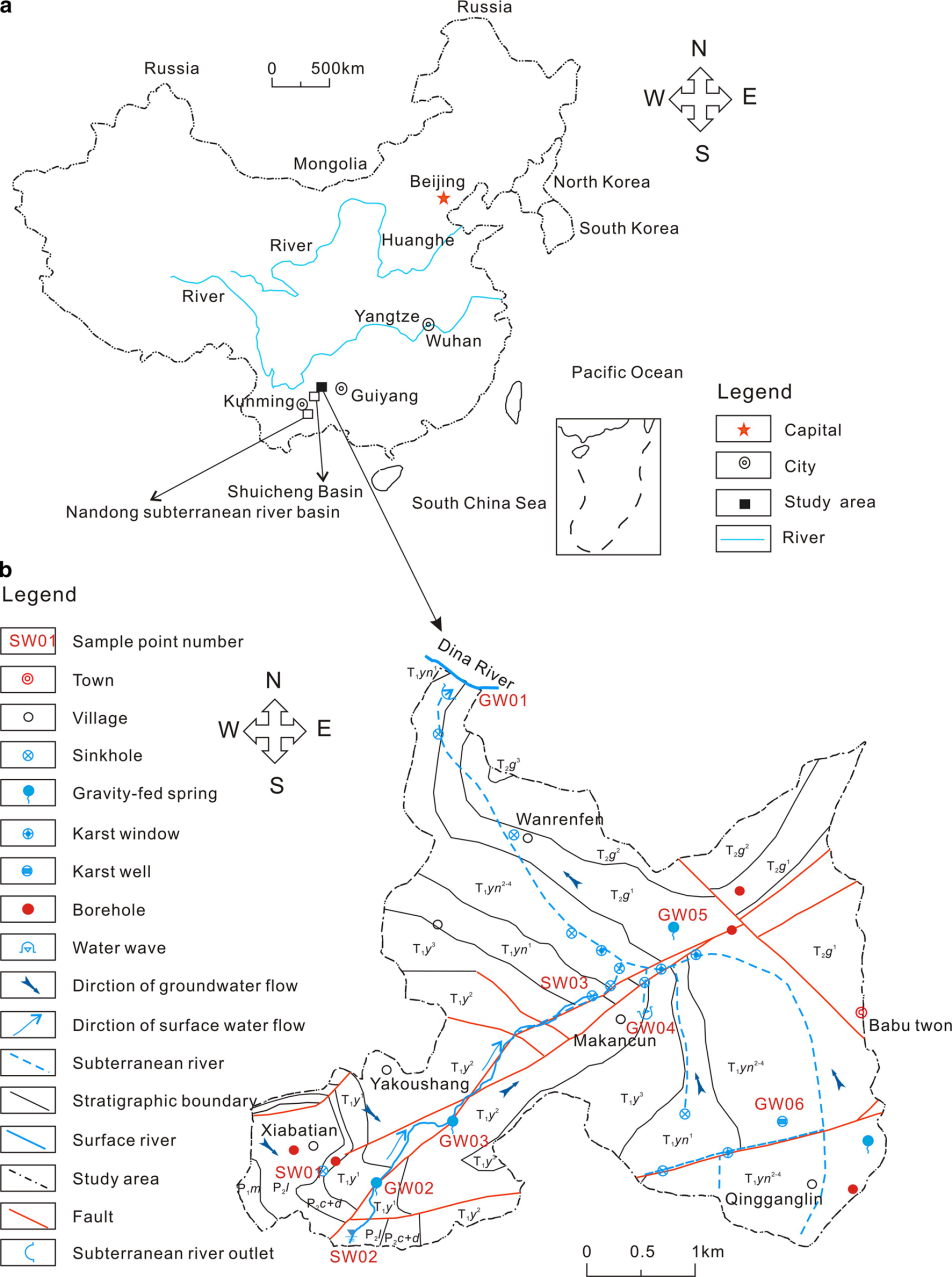
Location (**a**) and hydrogeological map and sampling site distribution (**b**) of the BSRB

**Fig. 2:**
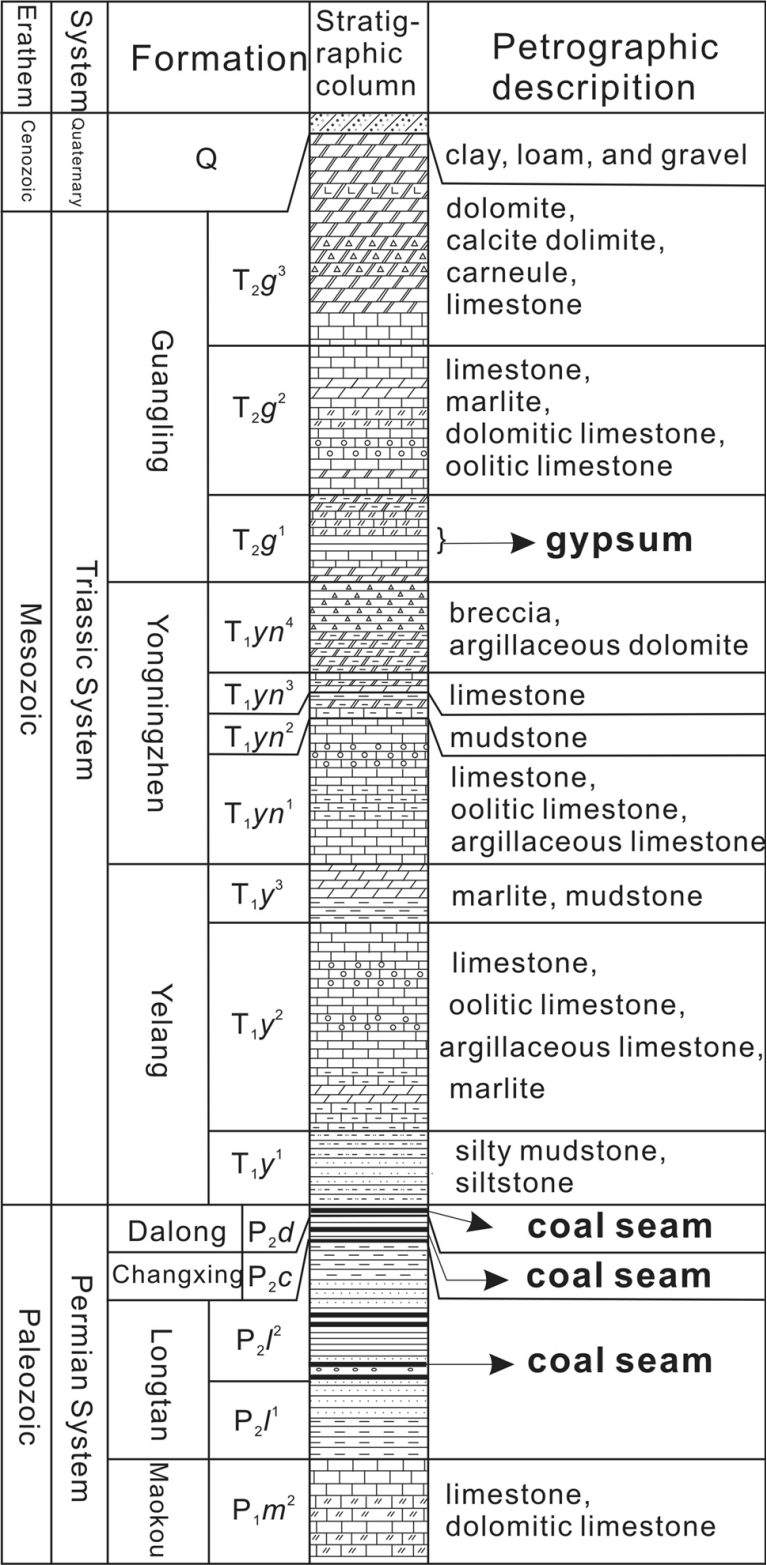
Stratigraphic column of the BSRB

The BSRB in the northeast of the Yunnan Guizhou Plateau covers an area of 18.08 km^2^. It is located between the north–south-trending tectonic zone of Sichuan and Guizhou and the north–south-trending tectonic zone of western Yunnan. The area has a mid-subtropical monsoon climate with a multiyear average annual temperature of 14.1 °C. The average annual precipitation is 1402.8 mm, 83.6% of which is concentrated mainly between May and October. The strata in this area are characterized by shallow-marine sediments of mostly Permian and Triassic age (Fig. 1), with a relatively thin Quaternary upper layer. The Permian and Triassic strata cover 1.17 and 16.91 km^2^, accounting for 6.47 and 93.53% of the total area, respectively. The Quaternary deposits consist of clay, loam, and gravel and they cover the bedrock. Figure 2 shows the lithological information obtained from five boreholes. The carbonate aquifer group is distributed most widely, covering an area of 14.96 km^2^, which accounts for 82.7% of the total area. The clastic aquifer group occupies only 3.12 km^2^, accounting for 17.3% of the total area. The studied basin is a bare karst area where carbonates provide the necessary physical conditions for karst development and where sinkholes, karst windows, and karst caves have developed. The subterranean river investigated in the present study is located upstream of the Wujiang River and it belongs to the Yangtze River system. It runs from southeast to northwest into the Dina River.

The BSRB belongs to the administrative district of Zhijin County in Guizhou Province. The area has a thin and barren soil layer, fragile ecological environment, and it is sparsely populated with only 10–20 resident households. Crops planted within the area comprise mainly rice and corn; however, in order to reduce costs, farmers rarely use fertilizers because of the frequent occurrence of both floods and droughts. There is no industrial activity within the study area except for a few coal mines.

## Sampling and analysis

Given the small area of the BSRS, ten representative water samples were collected in August 2014 with consideration of the water sources, recharge area, and lithology of the outcrops at the sampling sites. The samples included one rainwater sample (RW), three surface water samples (SW), and six groundwater samples (GW). The distribution of the sampling sites is shown in Fig. 1.

### Sampling

Water samples for conventional hydrochemical analyses of ions, $\delta {\text{D}}_{{{\text{H}}_{2} {\text{O}}}}$, and $\delta^{18} {\text{O}}_{{{\text{H}}_{ 2} {\text{O}}}}$ were collected using 50-mL polyethylene bottles. For cation analysis, super pure HNO_3_ (1:1) was added to the samples until a pH value of <2 was attained. For analyses of sulfate $\delta^{ 3 4} {\text{S}}_{{{\text{SO}}_{4} }}$ and $\delta^{ 1 8} {\text{O}}_{{{\text{SO}}_{4} }}$, the samples were collected using 2-L brown plastic bottles and super pure HCl was added to reach a pH value of <2. Subsequently, BaCl_2_ was added to precipitate all SO_4_^2−^ as BaSO_4_, which was then purified for further analysis using diethylenetriaminepentaacetic acid. After freezing, the obtained BaSO_4_ powder was sent to China University of Geosciences (Wuhan, China) for isotopic analysis. All water samples were filtered through a membrane filter (0.45-µm pore size) prior to collection and stored at 4 °C until analysis.

### Sample analysis

HCO_3_^−^ was titrated in the field using an alkalimeter with precision of 0.1 mmol L^−1^. The pH value of the water was determined in the field using a WTW Multi3430 (WTW Company, Germany) with precision of 0.01. Cations (Ca^2+^, Mg^2+^, Na^+^, and K^+^) were analyzed by atomic absorption spectrometry and anions (SO_4_^2−^, Cl^−^, and NO_3_^−^) were measured by high-performance liquid chromatography. Both $\delta {\text{D}}_{{{\text{H}}_{2} {\text{O}}}}$ and $\delta^{18} {\text{O}}_{{{\text{H}}_{ 2} {\text{O}}}}$ compositions were determined using a stable isotope mass spectrometer (MAT253, Thermo Fisher Scientific, USA) with precision greater than 0.1 and 0.05%, respectively. The $\delta^{ 3 4} {\text{S}}_{{{\text{SO}}_{4} }}$ and $\delta^{ 1 8} {\text{O}}_{{{\text{SO}}_{4} }}$ compositions were analyzed using an elemental analyzer (Carlo Erba 1108) combined with a stable isotope mass spectrometer (Delta V Advantage and MAT253) with precision greater than 0.2 and 0.05‰, respectively. Anions (SO_4_^2−^, Cl^−^, and NO_3_^−^), cations (Ca^2+^, Mg^2+^, Na^+^, and K^+^), and $\delta {\text{D}}_{{{\text{H}}_{2} {\text{O}}}}$ and $\delta^{18} {\text{O}}_{{{\text{H}}_{ 2} {\text{O}}}}$ compositions were analyzed at the Karst Geological Resources and Environment Supervision and Monitoring Center of the Ministry of Land and Resources.

## Results

### Hydrochemical characteristics

Table 1 shows the chemical compositions of rainwater, surface water, and groundwater in the BSRB. The level of total dissolved solids ranges from 352.88 to 933.19 mg L^−1^ (average: 588.49 mg L^−1^) in surface water and from 259.36 to 387.86 mg L^−1^ (average: 332.31 mg L^−1^) in groundwater. The rainwater has a pH value of 6.85, indicating that it is slightly acidic and belongs to the hydrochemical water type of SO_4_·HCO_3_–Ca. Among the three surface water samples, SW01 has the lowest pH (2.70) and its SO_4_^2−^ concentration is as high as 705.79 mg L^−1^. However, it does not reach a detectable level of HCO_3_^−^ and thus, it belongs to the hydrochemical water type of SO_4_–Ca·Mg. Samples SW02 and SW03 have a pH value of 7.23 and 7.69, respectively. In these two samples, Ca^2+^ is the dominant cation (>75% in terms of milligram equivalent per liter (meq L^−1^)) and [HCO_3_^−^ + SO_4_^2−^] are the dominant anions, but the proportion of [SO_4_^2−^] is higher than that of [HCO_3_^−^]. Therefore, the hydrochemical type of SW02 and SW03 is SO_4_·HCO_3_–Ca. The groundwater samples have pH values between 6.60 and 7.70 (average: 7.35); thus, they are considered slightly alkaline. In the groundwater samples, [Ca^2+^ + Mg^2+^] are the most dominant cations, accounting for >90% of the positive charges, while [K^+^] and [Na^+^] together account for <10%. [HCO_3_^−^ + SO_4_^2−^] are the dominant anions (>95%). In samples GW01 and GW04, the [HCO_3_^−^] concentrations are higher than those of [SO_4_^2−^] and their hydrochemical water type is HCO_3_·SO_4_–Ca. In samples GW02 and GW03, the [SO_4_^2−^] concentrations are higher than the [HCO_3_^−^] concentrations. [Cl^−^] accounts for <5% and their hydrochemical water type is SO_4_·HCO_3_–Ca. In GW05 and GW06, SO_4_^2−^ accounts for <20% of the total negative charges in meq L^−1^, while [Mg^2+^] accounts for >20% of the positive charges, resulting in a hydrochemical water type of HCO_3_–Ca·Mg. The concentrations of [K^+^ + Na^+^], [Cl^−^] and [NO_3_^−^] in rainwater, surface water, and groundwater in the BSRB are low; thus, do not play dominant roles among the cations and anions.

**Table 1:** Basic hydrochemical characteristics of rainwater, surface water, and groundwater in the BSRB

Sample ID	pH	TDS	K^+^	Na^+^	Ca^2+^	Mg^2+^	SO_4_^2−^	HCO_3_^−^	Cl^−^	NO_3_^−^	Outcropping stratum
		mg L^−1^									
Rain-water											
RW	6.85	–	0.09	0.38	6.98	0.24	12.36	9.31	1.38	–^a^	
Surface water											
SW01	2.70	933.19	3.97	9.45	92.14	32.80	705.79	0.00	2.79	0.94	P_2_ *l*
SW02	7.23	479.41	4.36	8.06	112.00	17.65	256.15	124.04	5.08	5.81	P_2_ *l*
SW03	7.69	352.88	1.83	5.17	90.76	12.53	150.10	144.71	2.76	9.88	T_1_ *yn* ^1^
Groundwater											
GW01	7.54	325.83	2.14	3.57	84.80	10.12	94.53	181.28	4.42	28.72	T_1_ *yn* ^2−4^
GW02	6.60	307.16	1.38	4.44	69.87	9.75	152.78	79.51	1.46	9.57	T_1_ *y* ^1^
GW03	7.27	353.39	1.42	4.50	93.80	9.71	136.72	154.25	3.29	18.40	T_1_ *y* ^2^
GW04	7.35	259.36	1.20	2.80	76.33	3.66	47.22	176.51	3.16	–	T_1_ *y* ^2−3^
GW05	7.70	360.26	1.91	1.70	69.68	40.22	58.33	338.71	4.27	2.00	T_2_g^1^
GW06	7.61	387.86	1.81	3.85	94.75	23.57	54.06	301.34	7.64	–	T_1_ *yn* ^2−4^

*TDS* Total dissolved solids

^a^No data

### Isotope values

Table 2 shows the $\delta {\text{D}}_{{{\text{H}}_{2} {\text{O}}}}$ values of the surface water samples vary between −45.7 and −32.1‰ (average: −40.47‰), while the $\delta^{18} {\text{O}}_{{{\text{H}}_{ 2} {\text{O}}}}$ values range from −7.19 to −5.01‰ (average: −6.33‰). For the groundwater samples, the $\delta {\text{D}}_{{{\text{H}}_{2} {\text{O}}}}$ values range between −56.2 and −46.4‰ (average: −52.62‰), and the $\delta^{18} {\text{O}}_{{{\text{H}}_{ 2} {\text{O}}}}$ values vary between −8.45 and −7.34‰ (average: −8.11 ‰).

**Table 2:** Isotope values (‰) of surface water and groundwater in the BSRB

Sample ID	$\delta D_{{{\text{H}}_{ 2} {\text{O}}}}$	$\delta^{18} {\text{O}}_{{{\text{H}}_{ 2} {\text{O}}}}$	$\delta^{ 3 4} {\text{S}}_{{{\text{SO}}_{4} }}$	$\delta^{ 1 8} {\text{O}}_{{{\text{SO}}_{4} }}$
Surface water				
SW01	−43.60	−6.79	−12.98	−0.54
SW02	−32.10	−5.01	−7.58	9.13
SW03	−45.70	−7.19	−10.91	5.40
Groundwater				
GW01	−52.50	−8.02	3.03	8.98
GW02	−51.60	−8.10	−14.32	2.81
GW03	−46.40	−7.34	−10.49	6.02
GW04	−54.70	−8.45	−6.80	6.72
GW05	−56.20	−8.37	16.58	14.35
GW06	−54.30	−8.40	−5.19	3.48

The $\delta^{ 3 4} {\text{S}}_{{{\text{SO}}_{4} }}$ values of the surface water samples range between −12.98 and −7.58‰ (average: −10.49‰), and the $\delta^{ 1 8} {\text{O}}_{{{\text{SO}}_{4} }}$ values vary between −0.54 and +9.13‰ (average: +4.66‰). For the groundwater samples, the $\delta^{ 3 4} {\text{S}}_{{{\text{SO}}_{4} }}$ values range between −14.32 and +16.58‰ (average: −2.87‰), and the *δ* ^18^O _SO4_ values vary between +2.81 and +14.35‰ (average: +7.06‰).

## Discussion

### Surface water and groundwater recharge sources

**Fig. 3:**
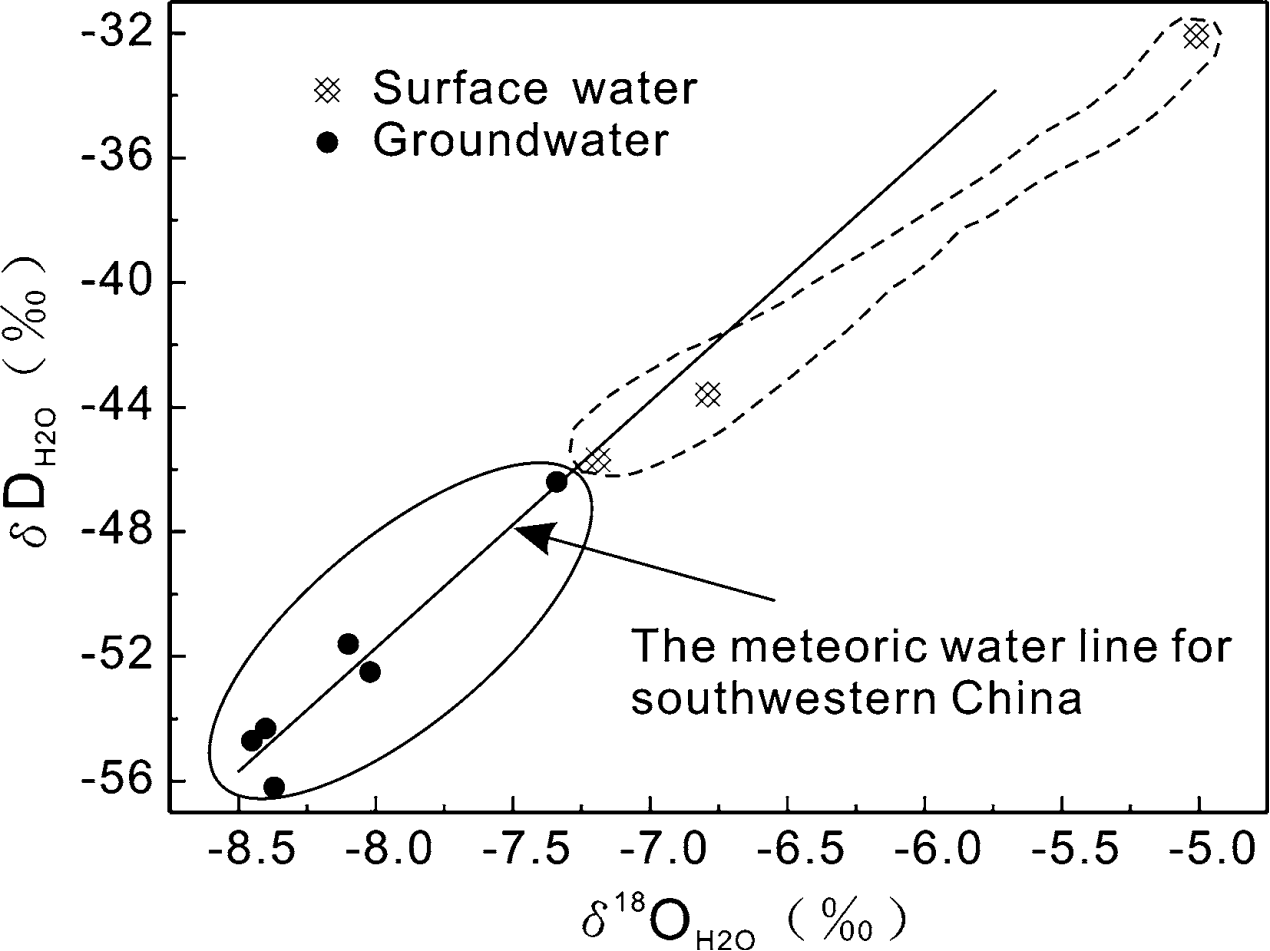
Relationship between *δ*D_H2O_ and *δ* ^18^O_H2O_ values in surface water and groundwater in the BSRB

Because of the rapid transformation between surface water and groundwater in karst areas, it is necessary to understand the local sources that replenish surface water and groundwater in order to explore further the sources of the components in these waters, particularly pollutants. Under low-temperature conditions, $\delta {\text{D}}_{{{\text{H}}_{2} {\text{O}}}}$ and $\delta^{18} {\text{O}}_{{{\text{H}}_{ 2} {\text{O}}}}$ compositions of water do not change through water–rock interactions [22]; thus, they are used widely to identify groundwater or mixed water recharge sources [23–26]. Craig [27] presented an equation for the relationship between $\delta {\text{D}}_{{{\text{H}}_{2} {\text{O}}}}$ and $\delta^{18} {\text{O}}_{{{\text{H}}_{ 2} {\text{O}}}}$, which has become known as the global meteoric water line: $\delta {\text{D}}_{{{\text{H}}_{ 2} {\text{O}}}} = 8.0\delta^{18} {\text{O}}_{{{\text{H}}_{ 2} {\text{O}}}} + 10.0$. Zhao et al. [28] proposed an equation for the relationship between $\delta {\text{D}}_{{{\text{H}}_{2} {\text{O}}}}$ and $\delta^{18} {\text{O}}_{{{\text{H}}_{ 2} {\text{O}}}}$ for southwestern China: $\delta {\text{D}}_{{{\text{H}}_{ 2} {\text{O}}}} = 7.96^{18} {\text{O}}_{{{\text{H}}_{ 2} {\text{O}}}} + 9.52$. Figure 3 shows that the $\delta {\text{D}}_{{{\text{H}}_{2} {\text{O}}}}$ and $\delta^{18} {\text{O}}_{{{\text{H}}_{ 2} {\text{O}}}}$ values of the surface water and groundwater samples plot close to the meteoric water line for southwestern China, indicating that the source of surface water and groundwater in the BSRB is atmospheric precipitation. Surface runoff or lake water resulting from precipitation is typically affected to a certain extent by evaporation, whereas precipitation that infiltrates directly through sinkholes or penetrates into an underground aquifer through the soil layers is not strongly affected by evaporation. Therefore, the $\delta {\text{D}}_{{{\text{H}}_{2} {\text{O}}}}$ and $\delta^{18} {\text{O}}_{{{\text{H}}_{ 2} {\text{O}}}}$ values of the surface water samples are higher than the groundwater samples (Fig. 3). This is consistent with results reported by both Pu et al. [20] and Yang et al. [29] from the Lijiang River Basin on the northwestern Yunnan Guizhou plateau and the Qingmuguan subterranean river in the eastern Sichuan Basin, respectively (both located in the karst area of southwestern China).

### Contribution of sulfuric acid to dissolution of carbonate rocks

Karstification occurs in an unbalanced solid–liquid–gas open system and it is a dynamic process involving the CO_2_–H_2_O–Ca^2+^ equilibrium. When only CO_2_ is involved in the dissolution of carbonate rocks, the following relationship between dissolved cations and HCO_3_^−^ is observed: [Ca^2+^ + Mg^2+^]:[HCO_3_^−^] = 1:1. The dissolution reaction can be written as follows:

1 $$ {\text{Ca}}_{x} {\text{Mg}}_{(1 - x)} {\text{CO}}_{3} + {\text{ H}}_{ 2} {\text{O }} + {\text{ CO}}_{2} = x{\text{Ca}}^{2 + } + \, \left( {1 - x} \right){\text{Mg}}^{2 + } + \, 2{\text{HCO}}_{3}^{ - } . $$

**Fig. 4:**
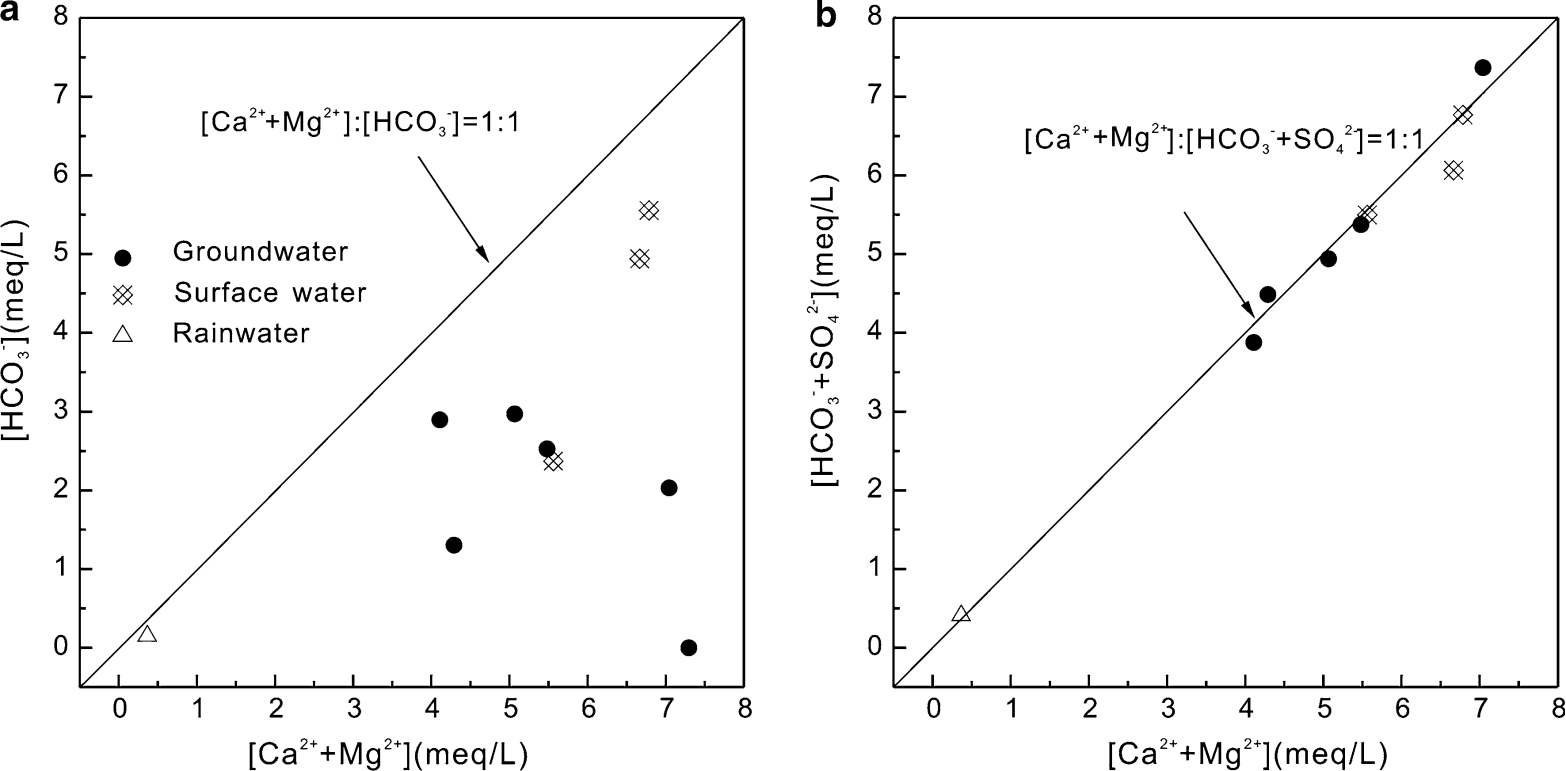
Relationship between [Ca^2+^ + Mg^2+^] and [HCO_3_^−^] (**a**) and [HCO_3_^−^ + SO_4_^2−^] (**b**) in rainwater, surface water, and groundwater in the BSRB

The compositional relationship of the dominant cations [Ca^2+^ + Mg^2+^] and the dominant anion [HCO_3_^−^] in the surface water and groundwater samples from the BSRB deviates from the [Ca^2+^ + Mg^2+^]:[HCO_3_^−^] = 1:1 equivalence line, with the samples plotting on the right side of the line (Fig. 4a). This indicates that other acids in both the surface water and groundwater are involved in the dissolution of carbonate rocks. Previous research has shown that sulfuric acid derived from natural processes and human activities can contribute to dissolution of carbonate rocks [30]. When sulfuric acid is present, the dissolution reaction can be written as follows:

2 $$ 3{\text{Ca}}_{x} {\text{Mg}}_{(1 - x)} {\text{CO}}_{ 3} + {\text{ H}}_{ 2} {\text{SO}}_{ 4} + {\text{ H}}_{ 2} {\text{CO}}_{3} = \, 3x{\text{Ca}}^{2 + } + \, 3\left( {1 - x} \right){\text{Mg}}^{2 + } + {{\text{ SO}}_{4}}^{2 - } + \, 4{\text{HCO}}_{3}^{ - } . $$

As shown in Eq. (2), when sulfuric and carbonic acid jointly participate in the dissolution of carbonate rocks, the following relationship between dissolved cations and anions is observed: [Ca^2+^ + Mg^2+^]:[HCO_3_^−^ + SO_4_^2−^] = 1:1. The compositional relationship of the dominant cations [Ca^2+^ + Mg^2+^] and the dominant anions [HCO_3_^−^ + SO_4_^2−^] in the surface water and groundwater samples from the BSRB are both at or close to the [Ca^2+^ + Mg^2+^]:[HCO_3_^−^ + SO_4_^2−^] = 1:1 equivalence line (Fig. 4b). This suggests that both sulfuric and carbonic acid participate in the dissolution of carbonate rocks in the BSRB, and that dissolution of carbonate rocks is the main source of Ca^2+^, Mg^2+^, and HCO_3_^−^ in both surface water and groundwater.

Ca^2+^ and Mg^2+^ in rainwater are derived mainly from weathering of carbonate rocks and Ca/Mg-containing particles produced by cement industries [31]. The [Ca^2+^ + Mg^2+^]:[HCO_3_^−^] values for the rainwater sample also deviate from the 1:1 equivalence line, plotting on the right side of the line (Fig. 4a). When [SO_4_^2−^] is considered, the [Ca^2+^ + Mg^2+^]:[HCO_3_^−^ + SO_4_^2−^] value lies on the 1:1 equivalence line (Fig. 4b), indicating that sulfuric acid participates in the dissolution of Ca/Mg-containing particles.

### SO_4_^2−^ concentrations in rainwater, surface water, and groundwater

Li et al. [9] reported that in the neighboring Shuicheng Basin (Fig. 1), SO_4_^2−^ concentrations of 63.1–110 mg L^−1^ (average: 84.24 mg L^−1^) were measured in surface water samples (*n* = 5). In ground water, the concentrations were 30–61.1 mg L^−1^, with an average of 45.55 mg L^−1^ (*n* = 2). For the Nandong subterranean river basin in Yunan Province, Jiang [32] reported concentrations of SO_4_^2−^ of 4.0–5.2 mg L^−1^ (average: 4.5 mg L^−1^) in rainwater (*n* = 3), 46.8–72.6 mg L^−1^ (average: 57.66 mg L^−1^) in surface water (*n* = 7), and 1.3–91.4 mg L^−1^ (average: 32.7 mg L^−1^) in groundwater (*n* = 36). The BSRB has SO_4_^2−^ concentrations of 12.36 mg L^−1^ in rainwater (*n* = 1), 150.1–705.79 mg L^−1^ (average: 370.68 mg L^−1^) in surface water (*n* = 3), and 47.22–152.78 mg L^−1^ (average: 90.61 mg L^−1^) in groundwater (*n* = 6). In comparison with the adjacent Shuicheng and Nandong subterranean river basins, SO_4_^2−^ is enriched more significantly in the precipitation, surface water, and groundwater samples in the BSRB.

**Fig. 5:**
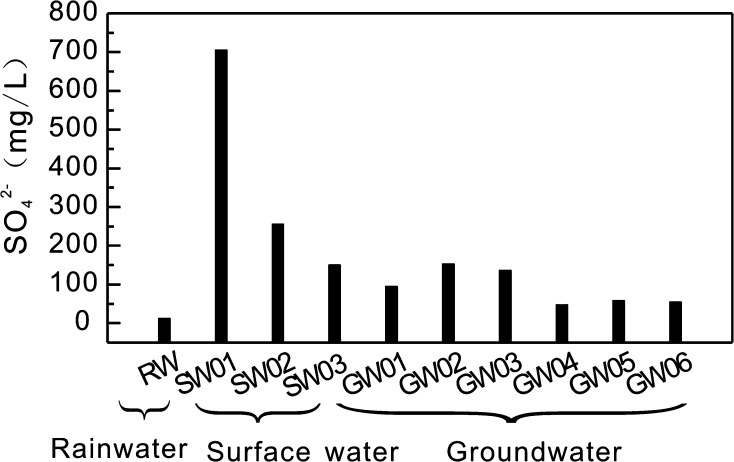
SO_4_^2−^ concentrations of precipitation, surface water, and groundwater in the BSRB

**Fig. 6:**
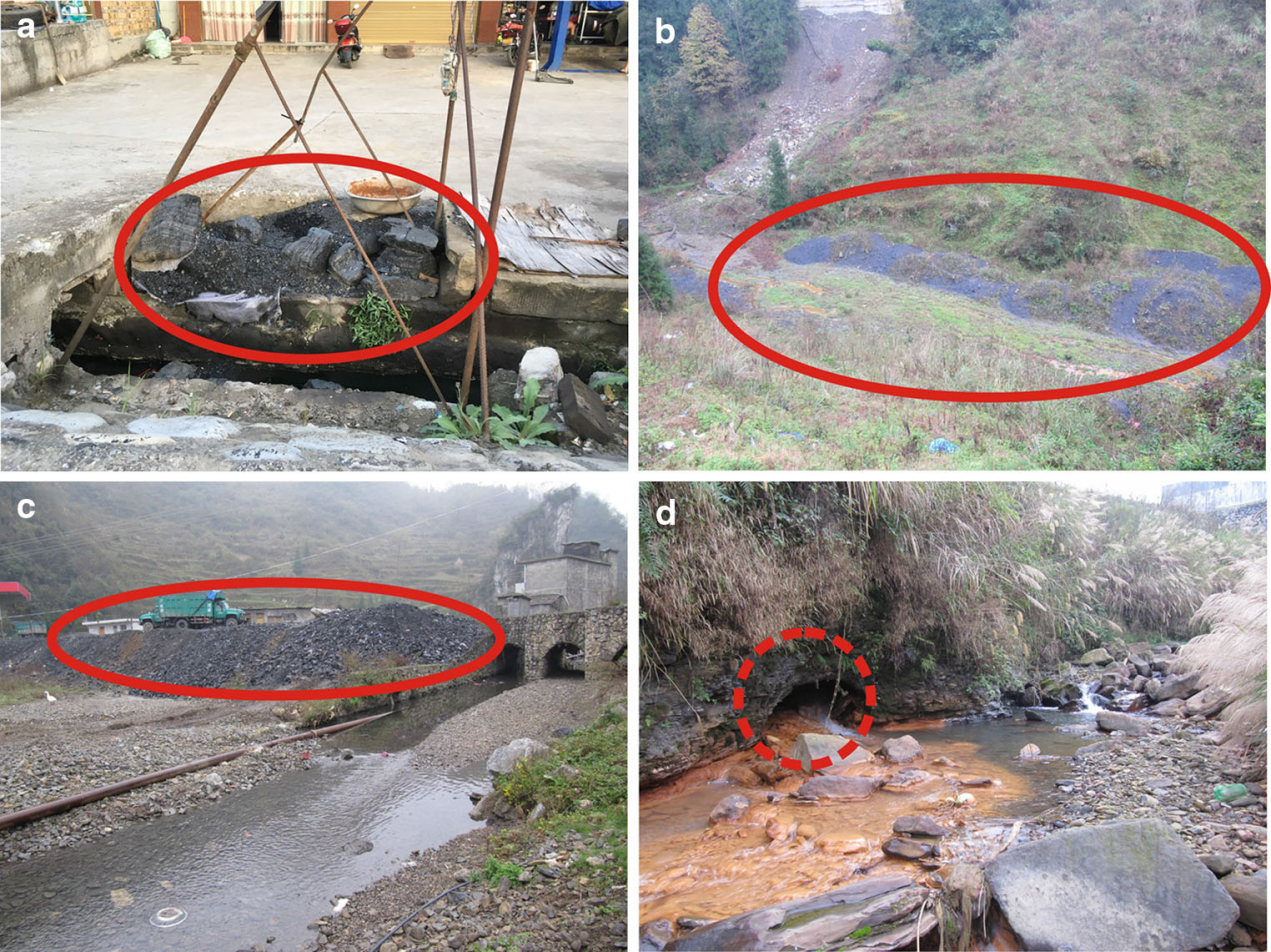
Coal stockpiled outside a farmer’s houses (**a**), low-grade coal stored in a karst depression (**b**), low-grade coal stored near a surface river (**c**), and water from an abandoned coal mine in the BSRB (**d**)

Figure 5 shows the concentrations of SO_4_^2−^ in rainwater, surface water, and groundwater in the BSRB. Overall, the order of SO_4_^2−^ concentration in the different samples is surface water > groundwater > rainwater. In the BSRB, farmers use coal as their primary energy source and they usually stockpile the coal outside their houses (Fig. 6a), whereas low-grade coal is generally stored arbitrarily at coal mines (Fig. 6b, c). In addition, the rainy season is usually characterized by heavy precipitation in this area. Consequently, coal leachates and water from abandoned coal mines (Fig. 6d) flow directly into the surface rivers, leading to high SO_4_^2−^ concentrations in the surface water.

Although the karst aquifer hinders the removal of SO_4_^2−^ in groundwater [33], it can adsorb SO_4_^2−^ to some extent [34]. In this study, the SO_4_^2−^ concentrations of the two gravity-fed spring samples (GW02 and GW03), which are located in the same water-conducting fracture zone (Fig. 1, GW02 is above the water flow of GW03), decrease by approximately 10.5% from 152.78 mg L^−1^ in GW02 to 136.72 mg L^−1^ in GW03 (Table 1). This is consistent with the findings by Guo et al. [34] and it indicates that SO_4_^2−^ concentrations could decrease, even when runoff water infiltrates directly into the karst aquifer through sinkholes, karst windows, and karst fissures. In addition, the subterranean river might have a certain dilution effect. Therefore, the SO_4_^2−^ concentration in groundwater is lower than in surface water. Precipitation is formed mainly from evaporated moisture that has relatively few impurities such as dissolved SO_2_ and sulfur-containing aerosols. Accordingly, the SO_4_^2−^ concentration of rainwater is lower than surface water and groundwater. The [SO_4_^2−^]:[HCO_3_^−^] ratio in the water of the BSRB is consistent with that in Guiyang City [35], indicating high diversity of SO_4_^2−^ sources in the researched region.

### Sources of SO_4_^2−^

#### Potential sources of SO_4_^2−^ in surface water and groundwater

Because of excessive mining and consumption of coal in Guizhou Province, Guizhou has become one of the provinces in southern China most affected by sulfuric acid rain [36]. Atmospheric precipitation, which is the main source for replenishing both surface water and groundwater in the BSRB, has a concentration of SO_4_^2−^ as high as 12.36 mg L^−1^. Therefore, precipitation is an important source of SO_4_^2−^ in both surface water and groundwater in this area. Figure 2 shows there are rich coal seams between the P_2_ *l*, P_2_ *c*, and P_2_ *d* strata in the BSRB, while the T_2_ *g* ^1^ stratum contains gypsum. The oxidation of sulfides in coal seams and the dissolution of gypsum would increase the concentration of SO_4_^2−^ in both surface water and groundwater [2, 17, 37]. The two reactions can be written as follows:

3 $$ {\text{FeS}}_{2} + {\text{ 15/4O}}_{ 2} + {\text{ 7/2H}}_{ 2} {\text{O }} = {\text{ Fe}}\left( {\text{OH}} \right)_{3} + \, 2{{\text{SO}}_{4}}^{2 - } + \, 4{\text{H}}^{ + } , $$

4 $$ {\text{CaSO}}_{ 4} = {\text{ Ca}}^{2 + } + {{\text{ SO}}_{4}}^{2 - } . $$

Jiang [38] found that sulfate contents in the yellow soil and lime soil of the Wujiang River Basin were very low, and that only very small amounts of SO_4_^2−^ enter the surface water and groundwater from these soils. This area has a very fragile ecological environment with relatively little human activity and limited use of agricultural chemical fertilizers. Hence, the amount of SO_4_^2−^ introduced by fertilizers, domestic sewage, and industrial wastewater is negligible. Therefore, the potential SO_4_^2−^ sources for surface water and groundwater in the BSRB, which has a comparable environment and level of human activities, are mainly precipitation, oxidation of sulfides in coal seams, and gypsum dissolution.

#### Source identification of SO_4_^2−^ in surface water and groundwater

The $\delta^{ 3 4} {\text{S}}_{{{\text{SO}}_{4} }}$ signature of atmospheric precipitation is not correlated with the SO_4_^2−^ concentration or the amount of precipitation, but it is related only to pollution sources [39]. The $\delta^{ 3 4} {\text{S}}_{{{\text{SO}}_{4} }}$ value of precipitation differs significantly between northern and southern China. In areas south of the Yangtze River, a larger amount of isotopically lighter sulfur is present in precipitation, resulting in negative $\delta^{ 3 4} {\text{S}}_{{{\text{SO}}_{4} }}$ values, whereas in areas to the north, mainly isotopically heavier sulfur is present, resulting in positive $\delta^{ 3 4} {\text{S}}_{{{\text{SO}}_{4} }}$ values [40]. The $\delta^{ 3 4} {\text{S}}_{{{\text{SO}}_{4} }}$ value in precipitation during summer in Guiyang City varies between −8.1 and −4.9‰ [40, 41]. Currently, in the Yangtze River Basin area, $\delta^{ 1 8} {\text{O}}_{{{\text{SO}}_{4} }}$ values in precipitation have been reported only for Wuhan City, which range between +8 and +15‰ [42]. Therefore, ranges of −8.1 to −4.9‰ and +8 to +15‰ were used as the $\delta^{ 3 4} {\text{S}}_{{{\text{SO}}_{4} }}$ and $\delta^{ 1 8} {\text{O}}_{{{\text{SO}}_{4} }}$ eigenvalues, respectively, to determine the fraction of SO_4_^2−^ that originates from atmospheric precipitation in this study.

Guizhou is a multi-age coal area. The upper Permian coal-bearing stratum contains the largest amount of coal and thus, it has become the main coal seam for mining because of its multiple advantages such as large reserves, shallow burial depth, and good exploration conditions. Guizhou coal is characterized by high sulfur content and a low $\delta^{ 3 4} {\text{S}}_{{{\text{SO}}_{4} }}$ value. It is a typical high-sulfur-content coal, as evidenced by sulfur contents ranging between 3.12 and 9.08% (average: 5.5%). The $\delta^{ 3 4} {\text{S}}_{{{\text{SO}}_{4} }}$ values vary between −15 and −2.51‰, with an average value of −7.52‰ [38, 40]. The $\delta^{ 1 8} {\text{O}}_{{{\text{SO}}_{4} }}$ signature resulting from oxidation of sulfides in the coal seams depends on the source of the oxygen for oxidation [43], and the $\delta^{ 1 8} {\text{O}}_{{{\text{SO}}_{4} }}$ values of Guizhou coal vary between −5 and +4‰ [14]. Therefore, ranges of −15 to −2.51‰ and −5 to +4‰ were used as the $\delta^{ 3 4} {\text{S}}_{{{\text{SO}}_{4} }}$ and $\delta^{ 1 8} {\text{O}}_{{{\text{SO}}_{4} }}$ eigenvalues, respectively, to determine the fraction of SO_4_^2−^ that originates from oxidized sulfides in the coal seams.

The gypsum in the Cambrian gypsolytes has the highest $\delta^{ 3 4} {\text{S}}_{{{\text{SO}}_{4} }}$ values of up to +32‰. The gypsum in the Permian and Triassic gypsolytes has relatively lower $\delta^{ 3 4} {\text{S}}_{{{\text{SO}}_{4} }}$ values, ranging between +10‰ and +28‰ [44], which is still higher than the values of the precipitation and the sulfides in the coal seams. The $\delta^{ 1 8} {\text{O}}_{{{\text{SO}}_{4} }}$ values of gypsum are also high, ranging between +14.5 and +32.5‰ [15, 16]. The BSRB consists mainly of Permian and Triassic strata, but it does not contain Cambrian strata. Therefore, ranges of +10 to +28‰ and +14.5 to +32.5‰ were used as the $\delta^{ 3 4} {\text{S}}_{{{\text{SO}}_{4} }}$ and $\delta^{ 1 8} {\text{O}}_{{{\text{SO}}_{4} }}$ eigenvalues, respectively, to identify SO_4_^2−^ that originates from gypsum dissolution.

**Fig. 7:**
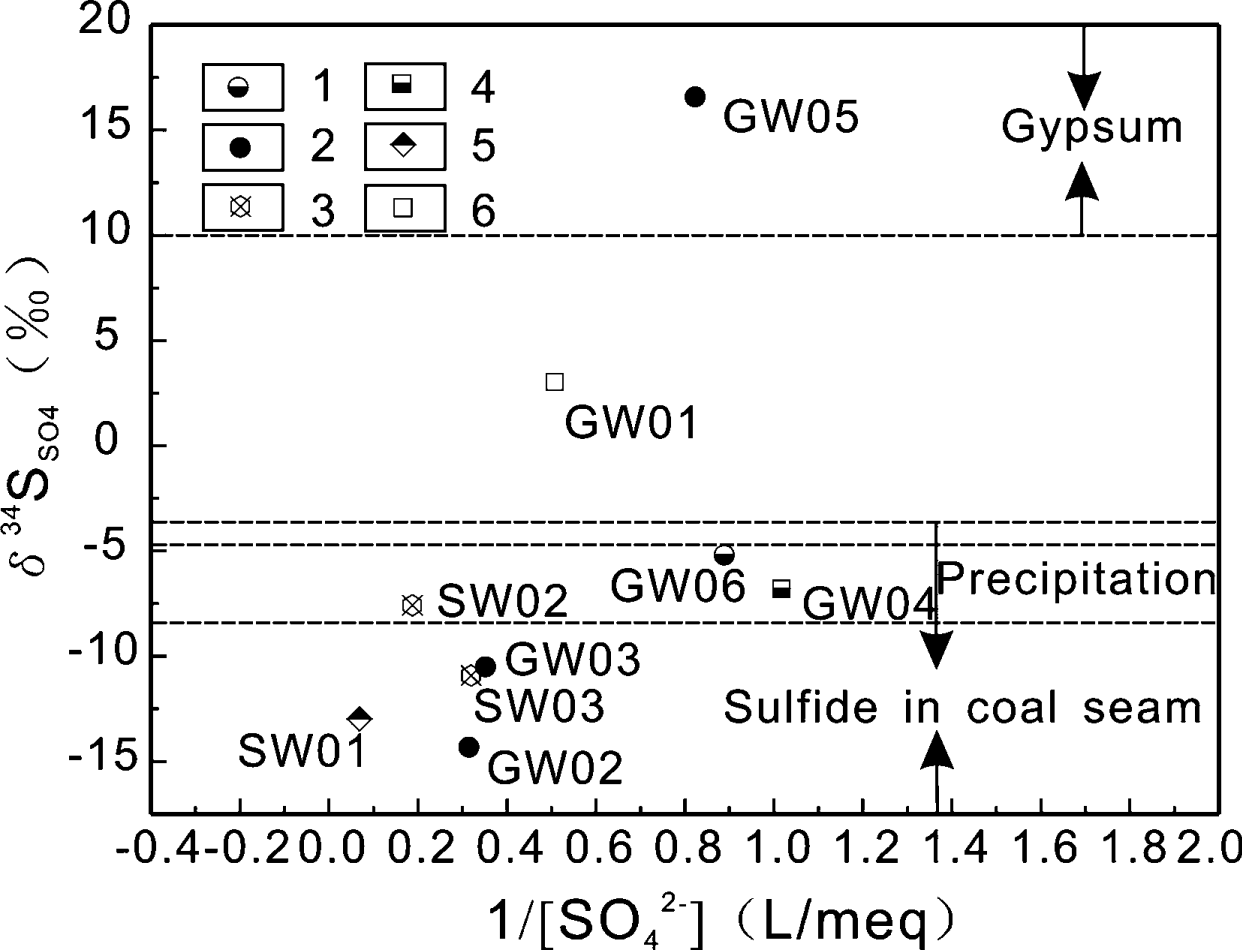
Relationship between $\delta^{ 3 4} {\text{S}}_{{{\text{SO}}_{4} }}$ and 1/[SO_4_^2−^] for different water types in the BSRB: *1* Karst wells; *2* gravity-fed springs; *3* surface water; *4* water discharging from karst caves; *5* coal mine pit water; *6* exit of the subterranean river

According to the relationship of $\delta^{ 3 4} {\text{S}}_{{{\text{SO}}_{4} }}$ and 1/[SO_4_^2−^] shown in Fig. 7, it is clear that the $\delta^{ 3 4} {\text{S}}_{{{\text{SO}}_{4} }}$ value of GW05 points toward dissolved gypsum as the SO_4_^2−^ source, and that samples SW01, SW03, GW02, and GW03 indicate sulfide oxidation in coal seams as the SO_4_^2−^ source. Because of the overlap of the $\delta^{ 3 4} {\text{S}}_{{{\text{SO}}_{4} }}$ values in precipitation and oxidized sulfides, SO_4_^2−^ sources for samples SW02, GW04, and GW06 cannot be identified accurately. In addition, the $\delta^{ 3 4} {\text{S}}_{{{\text{SO}}_{4} }}$ value of GW01 is different from the isotope ranges of all three potential sulfur sources. Sample GW05 was collected from a gravity-fed spring that outcrops in the T_2_ *g* ^1^ stratum (Fig. 1). Because this stratum contains gypsum (Fig. 2), the dissolution of gypsum is the main source of SO_4_^2−^ in GW05. Sample SW01 is pit water from an abandoned coal mine and it has a low pH value of 2.70, SO_4_^2−^ concentration of 705.79 mg L^−1^, and $\delta^{ 3 4} {\text{S}}_{{{\text{SO}}_{4} }}$ value of −12.95‰. The $\delta^{ 3 4} {\text{S}}_{{{\text{SO}}_{4} }}$ value of SW01 is close to the average value of −13‰ (*n* = 5) for coal mine wastewater in Guizhou, reported by Jiang et al. [38]. Sample SW03 is a water sample from a surface stream formed by water seeping through coal piles near residential areas (the distance between the sampling site and the coal pile is approximately 350 m). The stream water dissolves the underlying carbonate rocks, causing the pH value of the water to increase to 7.69. The $\delta^{ 3 4} {\text{S}}_{{{\text{SO}}_{4} }}$ value of SW03 is still −10.91‰, implying that the SO_4_^2−^ source might be oxidized sulfides from coal seams. Samples GW02 and GW03 are from two gravity-fed springs, both of which outcrop in the T_1_ *y* stratum (Fig. 2, this stratum does not contain coal seams) and are located in the same water-conducting fracture zone (Fig. 1). Groundwater from coal-rich zones flows to the sites of GW02 and GW03 and then emerges on the surface, with $\delta^{ 3 4} {\text{S}}_{{{\text{SO}}_{4} }}$ values of −14.32 and −10.49‰, respectively, indicating that SO_4_^2−^ originates from oxidized sulfides. Sample GW01 is water from the exit of the Babu subterranean river. Precipitation enters the subterranean river through sinkholes, karst windows, or by infiltration through the soil layer. During infiltration, the water passes through layers containing coal or gypsum and consequently, the SO_4_^2−^ in the subterranean river originates from different sources. By analyzing the $\delta^{ 3 4} {\text{S}}_{{{\text{SO}}_{4} }}$ composition of the water samples, we accurately identified that the source of SO_4_^2−^ in GW05 is mainly gypsum, and that the SO_4_^2−^ in samples SW01, SW03, GW02, and GW03, is derived from coal seams containing sulfides. However, GW01 has mixed SO_4_^2−^ sources, namely precipitation, oxidation of sulfides in coal seams, and gypsum dissolution.

**Fig. 8:**
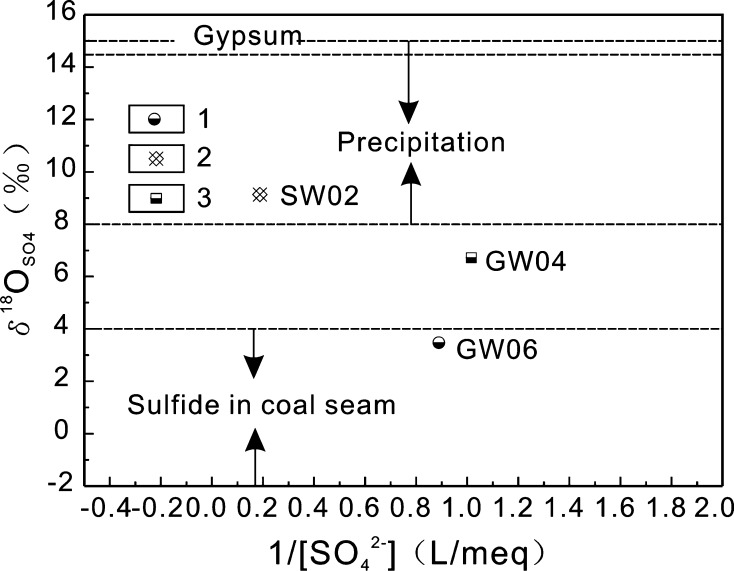
Relationship between $\delta^{ 1 8} {\text{O}}_{{{\text{SO}}_{4} }}$ and 1/[SO_4_^2−^] in different water types in the BSRB; *1* Karst wells; *2* surface water; *3* water-discharging karst cave

For sampling sites SW02, GW04, and GW06, $\delta^{ 3 4} {\text{S}}_{{{\text{SO}}_{4} }}$ analysis alone is insufficient for source identification of SO_4_^2−^ because of the overlapping $\delta^{ 3 4} {\text{S}}_{{{\text{SO}}_{4} }}$ ranges of precipitation and sulfides in coal seams; therefore, $\delta^{ 3 4} {\text{S}}_{{{\text{SO}}_{4} }}$ and $\delta^{ 1 8} {\text{O}}_{{{\text{SO}}_{4} }}$ need to be used jointly (Fig. 8). The site of SW02 is a small surface creek fed by precipitation, and the sampled water does not flow through coal seams or gypsum-containing strata (Fig. 1). Consequently, the $\delta^{ 1 8} {\text{O}}_{{{\text{SO}}_{4} }}$ value of sample SW02 is +9.13‰, which is within the eigenvalue range for SO_4_^2−^ of precipitation origin, i.e., between +8 and +15‰, suggesting that the main source of SO_4_^2−^ in SW02 is precipitation. The sampling site of GW04 is a water-discharging karst cave where water flows at a rate of 7.5 L s^−1^. Because of the wide potential source area, the water at site GW04 might come from coal seams or gypsum-bearing strata, leading to a $\delta^{ 1 8} {\text{O}}_{{{\text{SO}}_{4} }}$ value representing multiple SO_4_^2−^ sources. Sample GW06 is karst well water, which represents the maximum burial depth of the underground water. Its $\delta^{ 1 8} {\text{O}}_{{{\text{SO}}_{4} }}$ value falls within the range representing oxidized sulfides in coal seams as the source of SO_4_^2−^.

In summary, the main source of SO_4_^2−^ in surface water sample SW02 from the BSRB is precipitation. The main SO_4_^2−^ source for surface water samples SW01 and SW03 and underground water samples GW02, GW03, and GW06 is sulfide in coal seams. The main source of SO_4_^2−^ in GW05 is gypsum, while GW01 and GW04 have mixed SO_4_^2−^ sources.

### Contribution of different SO_4_^2−^ sources to the karst water system

The flux at the exit of the subterranean river represents the total water flow of the karst water system and it can provide information about the temporal and spatial distributions of water sources as well as the surface and underground water passages [45]. Therefore, the exit of the river is an important monitoring location for karst water. As discussed in the preceding section, the SO_4_^2−^ in the water at the exit of the Babu subterranean river originates from precipitation, coal seams, and gypsum. Therefore, the relative contributions of the three sources can be calculated based on the $\delta^{ 3 4} {\text{S}}_{{{\text{SO}}_{4} }}$ value of the water at the main outlet (GW01) using the formula below:

5 $$ \delta^{34} {\text{S}}_{{{\text{SO}}_{ 4}^{\text{ - GW01}} }} = x\delta^{34} {\text{S}}_{{{\text{SO}}_{ 4}^{\text{ - coal}} }} + (1 - x - y)\delta^{34} {\text{S}}_{{{\text{SO}}_{ 4}^{\text{ - gyp}} }} $$

where *x* (%) is the percentage of SO_4_^2−^ from precipitation, *y* (%) is the percentage of SO_4_^2−^ from coal seams, (1−*x*−*y*) (%) is the percentage of SO_4_^2−^ from gypsum dissolution, $\delta^{ 3 4} {\text{S}}_{{{\text{SO}}_{4}^{{ - {\text{GW01}}}} }}$ (‰) is the $\delta^{ 3 4} {\text{S}}_{{{\text{SO}}_{4} }}$ value of the water at the exit of the subterranean river, $\delta^{ 3 4} {\text{S}}_{{{\text{SO}}_{4}^{{ - {\text{rain}}}} }}$ (‰) is the $\delta^{ 3 4} {\text{S}}_{{{\text{SO}}_{4} }}$ value of the precipitation in the river basin, $\delta^{ 3 4} {\text{S}}_{{{\text{SO}}_{4}^{{ - {\text{coal}}}} }}$ (‰) is the $\delta^{ 3 4} {\text{S}}_{{{\text{SO}}_{4} }}$ value of the water sample representing oxidized sulfides in coal seams, and $\delta^{ 3 4} {\text{S}}_{{{\text{SO}}_{4}^{{ - {\text{gyp}}}} }}$ (‰) is the $\delta^{ 3 4} {\text{S}}_{{{\text{SO}}_{4} }}$ value of the water sample representing dissolssved gypsum in the river basin. In accordance with the mass conservation law, the contribution of precipitation is calculated to be 13%. In the calculations, an average $\delta^{ 3 4} {\text{S}}_{{{\text{SO}}_{4} }}$ value of −7‰ for summer precipitation in Guiyang City is used as the $\delta^{ 3 4} {\text{S}}_{{{\text{SO}}_{4} }}$ precipitation value [40]. The average $\delta^{ 3 4} {\text{S}}_{{{\text{SO}}_{4} }}$ value of SW01, SW03, GW02, GW03, and GW06 is used to represent the oxidized sulfides in the coal seams as the SO_4_^2−^ source. The $\delta^{ 3 4} {\text{S}}_{{{\text{SO}}_{4} }}$ value of sample GW05 is used to represent dissolved gypsum as the SO_4_^2−^ source. The calculation yields the contributions from sulfide oxidation in coal seams and gypsum dissolution are 40 and 47%, respectively. It is acknowledged that the calculation result might be affected by the small number of precipitation and surface water samples. However, the finding that the contribution from oxidized sulfides in coal seams is smaller than the contribution from gypsum dissolution is in accordance with the observation that coal seams (approximately 6.2% of the total area) occupy a smaller part of the study area than the gypsum-containing strata (approximately 17.1% of the total area).

The contribution of SO_4_^2−^ from precipitation to the Babu subterranean river water derived in this study is slightly smaller than that reported by both Li et al. [1] and Zhang et al. [12] for the Jialing and Yellow River areas, respectively. This might be attributable to a buffering effect during precipitation infiltration into the subterranean river or to chemical changes of the water flowing through the coal seams and gypsum strata. However, the SO_4_^2−^ contribution from precipitation to the subterranean river outflow reaches 13%, demonstrating that the adverse effect on underground water quality by acidic rain resulting from the consumption of coal by human activities cannot be overlooked. The open storage of coal also contributes to the large contribution of SO_4_^2−^ (40%) from sulfide oxidation in coal seams. Therefore, it is necessary to require local residents and coal mining companies to ensure coal is stored appropriately.

## Conclusions

In the BSRB area, the main source for surface water and groundwater is precipitation, and the main source of Ca^2+^, Mg^2+^, and HCO_3_^−^ in these waters is the dissolution of carbonate rocks. Together with carbonic acid, sulfuric acid contributes to the dissolution of carbonate rocks and Ca^2+^/Mg^2+^-containing particles produced by cement industries. In the study area, the concentration of SO_4_^2−^ in rainwater is 12.36, 150.1–705.79 mg L^−1^ (average: 70.68 mg L^−1^) in surface water, and 47.22–152.78 mg L^−1^ (average: 90.61 mg L^−1^) in groundwater. Accordingly, the order of SO_4_^2−^ concentration in the different samples is surface water > groundwater > rainwater. Compared with adjacent regions, the rainwater, surface water, and groundwater show SO_4_^2−^ enrichment in the BSRB. The $\delta^{ 3 4} {\text{S}}_{{{\text{SO}}_{4} }}$ and $\delta^{ 1 8} {\text{O}}_{{{\text{SO}}_{4} }}$ values in the surface water samples range between −12.98 and −10.19‰, and between −0.54 and +9.13‰, respectively. The main sources of SO_4_^2−^ are precipitation for SW02 and sulfide oxidation in coal seams for SW01 and SW03. The $\delta^{ 3 4} {\text{S}}_{{{\text{SO}}_{4} }}$ and $\delta^{ 1 8} {\text{O}}_{{{\text{SO}}_{4} }}$ values of the groundwater samples range between −14.32 and +16.58‰ and between +2.81 and +14.35‰, respectively. The main sources of SO_4_^2−^ are sulfide oxidation in coal seams for GW02, GW03, and GW06, and gypsum dissolution for GW05. At sampling sites GW01 and GW04, SO_4_^2−^ originates from mixed sources. The SO_4_^2−^ contribution of precipitation to the water at the exit of the Babu subterranean river is 13%; sulfide oxidation in coal seams contributes 40%, and gypsum dissolution contributes 47%. The mining, open storage, and consumption of coal have all exerted significant adverse impacts on the water quality of the Babu subterranean river that should not be overlooked. The BSRB and the entire province should develop a sustainable strategy for the exploration and use of coal in order to balance the needs of economic development and water quality protection.
